# Abdominal aortic injury caused by a sharp osteophyte displaced by a compression fracture: A case report and literature review

**DOI:** 10.1016/j.heliyon.2024.e35994

**Published:** 2024-08-08

**Authors:** Kazuyuki Miyamoto, Mako Sakakibara, Hiroki Yamaga, Atsuo Maeda, Masaharu Yagi, Kenji Dohi

**Affiliations:** a Department of Emergency, Critical Care and Disaster Medicine, Showa University School of Medicine, 1-5-8 Hatanodai, Shinagawa-ku, Tokyo, 142-8555, Japan; b Department of Emergency Medicine, Showa University Northern Yokohama Hospital, 35−1 Chigasaki Chuo Tsuzuki-ku, Yokohama City, 224-8503, Japan; c Department of Emergency, Critical Care Medicine, Showa University Fujigaoka Hospital, 1-30 Fujigaoka Aoba-ku, Yokohama City, 224-8503, Japan

**Keywords:** Osteophytes, Abdominal injury, Abdominal aorta, Fractures, Compression, Case reports

## Abstract

Osteophytes grow on vertebral bodies and sometimes form a sharp edge, which can induce traumatic abdominal aortic injury (TAAI). However, these cases are extremely rare, although osteophytes grow in almost everyone with age. Herein, we report a rare case of TAAI due to a sharp osteophyte dislocation following a compression fracture, with a literature review. An 83-year-old man presented with back pain after a fall and subsequently developed shock. His lactate level was elevated on arrival, although we could not identify the cause of the shock. Enhanced computed tomography (CT) revealed a compression fracture of the lumbar spine, a sharp osteophyte penetrating the abdominal aorta, and contrast media spurting to the retroperitoneum. We immediately transfused, administered continuous noradrenaline to maintain his vital signs, and consulted a vascular surgeon and radiologist to arrange for an emergency operation. However, the operating room was not immediately available. We tried to evacuate a trauma center, which took time due to the COVID-19 outbreak. Thereafter, the patient's condition deteriorated, resulting in a pulseless electrical activity, and he passed away 3 h after arrival. Afterward, the previous CT image and a sharp osteophyte were observed in the lumbar vertebrae in contact with the abdominal aorta. There were only eight reports where the aorta was injured by osteophytes. From our review, unlike the proportion of common TAAI, injuries due to strong external forces were relatively small. There was no specific symptom, and the vertebral levels of osteophytes were concentrated in Th12-L3. The most common morphology of aorta injuries is pseudoaneurysm formation. Moreover, five of the patients developed hemorrhage. Considering these previous reports, we should pay attention to the aorta in front of the vertebral bodies regardless of the mechanism of injury when a CT image of patients shows osteophytes.

## Highlights

• Lumbar disc degenerates with age and sometimes forms sharp and hard osteophytes.
• Anatomically, the abdominal aorta is located just anterior to the lumbar vertebra.
• Compression fracture displaced a sharp osteophyte and injured the abdominal aorta.

## 1 Introduction

Traumatic abdominal aortic injury (TAAI) is a rare but rapidly deteriorating hemodynamic condition, particularly if the diagnosis is delayed [1]. However, it is sometimes difficult to detect TAAI since extended focused assessment with sonography and radiography (chest and pelvis), which are required for the initial evaluation of trauma, cannot detect it. Therefore, it is usually suspected based on the mechanism of injury, including strong forces, such as acute deceleration forces in motor vehicle collisions in patients with hemodynamic instability following trauma [2]. The abdominal aorta is anatomically located in the retroperitoneal cavity and approximately close to the lumbar vertebrae. In old age, disc degeneration leads to osteoarthritis, which is often associated with osteophyte growth on the vertebral bodies. Osteophyte growth commonly occurs between the L3 and L4 vertebrae, and its growth is correlated with age [3]. Two types of osteophytes can form on the anterior aspects of the lumbar vertebrae: claw and traction types. Particularly, the claw type sometimes forms a sharp edge [4]. In addition, aging stiffens large arteries, which induces less elasticity and makes them more susceptible to direct external [5]. It injures the abdominal aorta when a sharp osteophyte is displaced by a compression fracture in patients with fragile bone. Hence, TAAI might occur without a strong external force, such as a fall occurring in old age. However, these cases are rare; including our cases, only eight were reported in the past. Herein, we report a rare case of TAAI due to a sharp osteophyte dislocation following a lumbar vertebral compression fracture after a fall. Moreover, we reviewed previous reports of aortic injuries due to osteophytes.

## 2 Case presentation

**Fig. 1:**
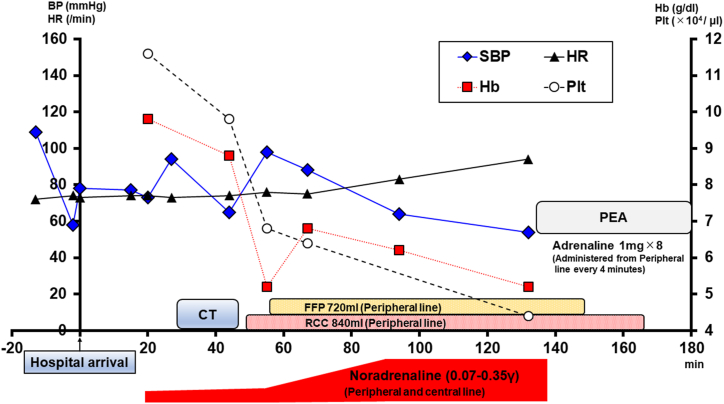
Progress chart of the patient BP; Blood pressure, CT; Computed tomography, FFP; Fresh frozen plasma, Hb; Hemoglobin, HR; Heart rate, PEA; Pulseless electrical activity, Plt; Platelet, RCC; Red cell concentrate, SBP; Systolic blood pressure.

**Fig. 2:**
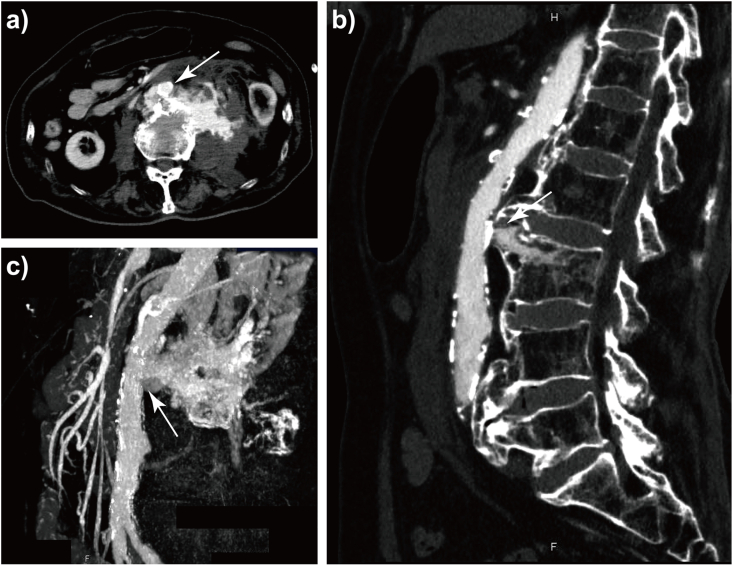
Enhanced-CT and angiography. (a) (b) The compression fracture of the lumbar vertebra (L3), with sharp osteophyte (white arrow), injured the abdominal aorta (c) Contrast media was spurting from the abdominal aorta (white arrow) into the retroperitoneum.

**Fig. 3:**
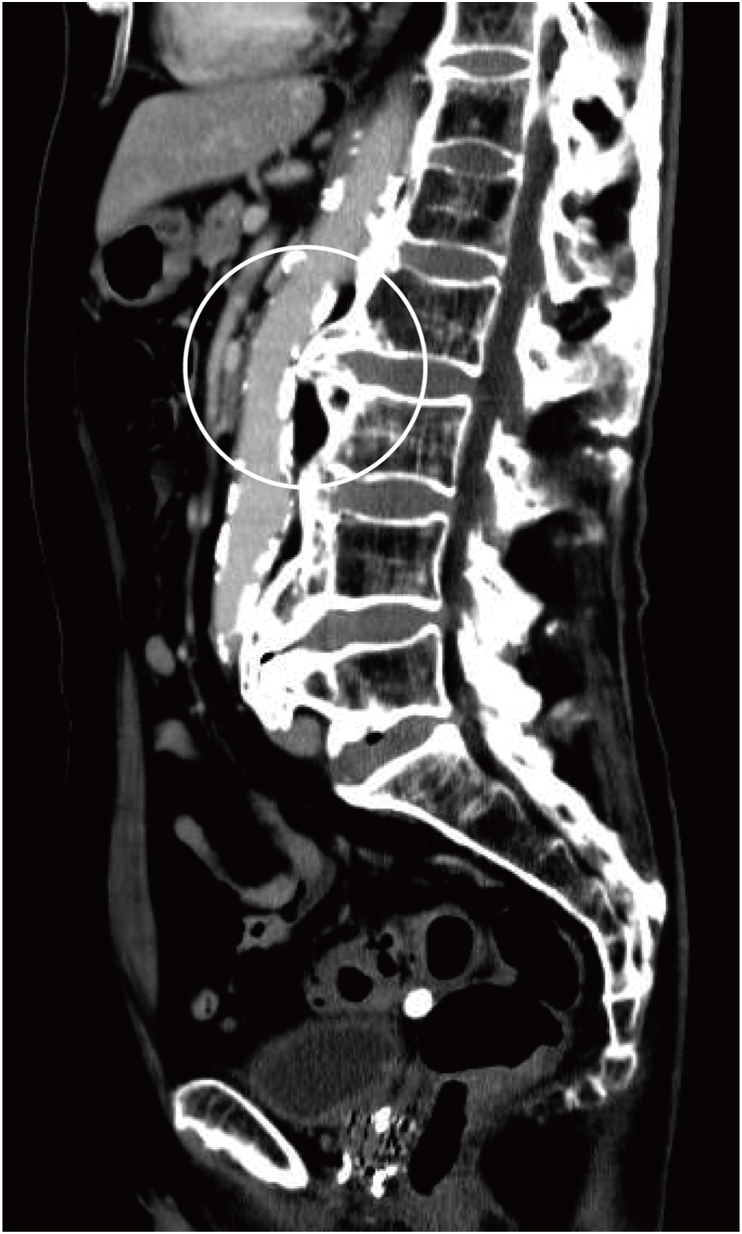
Sagittal section of CT, which was taken 2 years ago. A sharp osteophyte (white circle) in the lumbar spine (L 2/3), which was in touch with the abdominal aorta.

An 83-year-old man was transferred to the emergency department (ED) after stumbling and falling while shopping in the supermarket with his family's help. He had undergone brachytherapy for prostate carcinoma 10 years previously and had regular follow-ups at our hospital. He also had a history of several colonic diverticular hemorrhages and had been admitted to our facility several times. Regarding medications, he was taking pregabalin (150 mg/day). He was conscious and hemodynamically stable, with blood pressure (BP) of 109/58 mmHg, heart rate (HR) of 72/min, respiratory rate (RR) of 24 times/min, body temperature of 36.7 °C, and SpO_2_ of 100 % at room air, when the emergency medical service arrived. Therefore, he was transferred to our hospital, which is not a trauma center. Moreover, on the way to the ED, he complained of back pain and was in a state of shock just before arrival (Fig. 1). On arriving at ED, his BP was 78/52 mmHg, HR was 73/min, RR was 28 times/min, and his Glasgow Coma Scale score and his extremities were cold, which suggested a shock. We immediately infused crystalloid (Up to 2000 mL) and continuous noradrenaline (0.07–0.35 γ) while trying to identify the cause of the shock. Extended focused assessment with sonography of the trauma did not detect any fluid collection. The X-ray of the chest and pelvis, electrocardiography, quick echocardiography, and rectal examination neither revealed obvious abnormalities. The arterial blood gas and laboratory examinations could detect, in the short term, slightly decreased hemoglobin level (10.7 → 9.8 g/dL) and platelet count (12.2 → 11.6 × 10^4^/μL) compared with previous data. Otherwise, the lactate level was remarkably elevated to 4.76 mmol/L, suggesting the presence of shock. After resuscitation, he improved somewhat (BP 94/56 mmHg, HR 73/min). Therefore, we decided to perform contrast-enhanced computed tomography (CT), which was located away from the ED on the second floor of the hospital. Subsequent imaging showed a compression fracture of the lumbar spine (L3), with sharp osteophytes penetrating the abdominal aorta and contrast media spurting from the abdominal aorta into the retroperitoneum cavity (Fig. 2a–c). Thus, the patient was diagnosed with TAAI due to sharp osteophyte dislocation at the time of the lumbar compression fracture. We immediately prepared red cell concentrate (RCC) and fresh frozen plasma (FFP) and consulted a vascular surgeon and radiologist to arrange for an emergency operation. However, since both of them were performing other surgeries, they could not respond immediately. After coming back from a CT examination, he went into shock again. We performed rapid sequence intubation with midazolam (2 mg) and rocuronium (30 mg) and started transfusion (total RCC 840 mL, FFP 720 mL). The dose of continuous noradrenaline was also increased to maintain the BP. We considered inserting the resuscitative endovascular balloon occlusion of the aorta (REVOA), prepared for an obstetric hemorrhagic crisis to reduce the hemorrhage. However, it had just been used the previous day, and a new one was yet to be supplied. The laboratory data at that time showed a decrease in hemoglobin (5.2 g/dL) and platelet (6.8 × 10^4^/μL). After starting the transfusion, the patient improved somehow, with systolic BP and hemoglobin levels improving to 98 mmHg and 6.8 g/dL, respectively. Then, the vascular surgeon and radiologist called to inform us that the patients needed emergency endovascular surgery, though the operating room and their staff were not immediately available. Thus, we endeavored to evacuate to the closest trauma center; however, they were unable to accept the patient due to the COVID-19 outbreak. Therefore, we contacted several trauma centers that were located 1 h ride from our facility and finally found one to accept him. By that time, the patient systolic BP had decreased to around 50 mmHg despite the blood transfusion and continuous noradrenaline. The laboratory data during preparation showed that the Hb and platelet were 5.2 g/dL and 4.4 × 10^4^/μL, respectively. While we were preparing to evacuate, the patient underwent pulseless electrical activity (PEA). He immediately received resuscitation in accordance with the American Heart Association guideline 2020. Adrenaline (1 mg) was infused every 4 min. While undergoing resuscitation, we also explained his condition to his family. They requested that cardiopulmonary resuscitation be discontinued, considering his age and prior state of health. He passed away 3 h after arriving at the ED. Subsequently, during a conference, we reviewed the CT image that had been taken previously. The sagittal section revealed a sharp osteophyte in the lumbar spine (L 2/3) that was in contact with the abdominal aorta (Fig. 3).

## 3 Discussion

Osteophytes are bony protrusions covered with fibrocartilage that sometimes form sharp, hard edges [6]. Chanapa [7] analyzed 108 dried Thai lumbar spine specimens and reported that 97.2 % presented with lumber osteophyte, which became longer with age and was significantly greater in males than females. In our patient, a CT performed 2 years previously, when he was 81 years old, showed a sharp, long osteophyte that was in contact with the posterior wall of the abdominal aorta. We assumed that this sharp osteophyte was displaced after the compression fracture, which induced an abdominal aortic injury. Although it is common that in old age, people develop osteophytes, there were only eight reports [[8], [9], [10], [11], [12], [13], [14]] where these injured the aorta. Hence, we reviewed the literature using the Medline database to look for similar case reports of “aortic injury due to osteophytes,” using “aorta” and “osteophyte” or “bone spar” as keywords to clarify the clinical features (Table 1). The median age of the patients was 62 [53–75.5] years, and they included six males and two females. The causes of the injuries were high-energy trauma such as traffic accidents, minor trauma due to tumbling, or repeated minor injuries, and unknown in three, two, and three cases, respectively. Unlike the proportion of TAAI, injuries due to strong external forces were relatively small. The symptoms were not specific: back pain in three patients, abdominal pain in two patients, and gastrointestinal symptoms such as nausea in two patients. The vertebral levels with osteophytes were concentrated around Th12-L3, the thoracolumbar transition area (Th12) most susceptible to external forces, to the lumbar vertebrae (L3), where the frequency of osteophytes is highest. The morphology of aorta injuries included penetration in one patient, pseudoaneurysm in five patients, and aneurysm in two patients. Moreover, five of the patients had hemorrhage, four patients underwent endovascular treatment, and three underwent surgery. Considering these previous reports, we should pay attention to the aorta in front of the vertebral bodies (Th12-L3) regardless of the mechanism of injury when a CT image of patients shows an osteophyte.

**Table 1:** 

	Report, year	Age, gender	Cause	Symptoms	Vertebra	Diagnosis	Treatment
1	Miyamoto et al, 2023 (Current case)	83, M	Tumbling	Back pain	L3	Aorta perforation by sharp osteophytes	NA
2	Kontopodis et al., 2020	75, M	Unknown	Asymptomatic	L2	The osteophyte penetrated the posterior wall of the AN, which was found during the operation.	Operation
3	Myers et al., 2015	54, M	Heavy metal door fall	Abdominal tenderness with guarding	Th11/12	Harpoon-like osteophyte attached the posterior wall of AN	Endovascular repair
4	Afifi et al., 2015	65, F	Unknown	Stiffness of her neck and shoulders, Fever	NA	Mycotic PA rupture	Operation, Antibiotics
5	Machado et al., 2013	59, M	Unknown	Asymptomatic	L3/4	Harpoon-like osteophyte attached the posterior wall of AN	Endovascular repair
6	Vernon et al., 2014	77, M	Traffic accident	Back pain	Th12/L1	PA rupture with retroperitoneal hematoma	Endovascular repair
7	Dregelid et al., 2007	46, F	Repeated minor occupational trauma	Acute upper abdominal pain radiating to the back, nausea	L1	PA with a needle-thin perforation	Operation
8	Chtata et al., 2005	50, M	Traffic accident	NA	Th11/12	PA without rupture	Endovascular repair

AN; aneurysm, F; female, L; Lumber vertebrae, M; male, NA; Not available, Th; thoracic vertebrae, PA; pseudoaneurysm.

In our patient, the hemodynamics became unstable again after the CT. It is undeniable that the vertebral body movement caused by the transfer to the CT may have aggravated the hemorrhage. If we had inserted REVOA before leaving ED, considering the retroperitoneal hemorrhage (including the lumbar artery injury or renal injury), we could have promptly responded to the deterioration of hemodynamics with endovascular treatment.

The advantage of our report was that we were able to compare the CT images before and at the time of injury, which revealed how the abdominal aorta was injured by the dislocation of the sharp osteophyte associated with the compression fracture. There were no other reports that compared pre-injury and injury images. However, the case report had some limitations. First, the patient died. Secondly, the number of similar cases identified in our mini-review was small, and our description was therefore limited. Hence, further reports will be needed to clarify the clinical features of TAAI by sharp osteophytes.

## 4 Conclusion

Falls are one of the most common mechanisms of injury among older people [1]. Although it is rare, the abdominal aorta can be injured by the sharp osteophyte. Hence, attention should be paid to the aorta in front of the vertebral bodies, regardless of the mechanism of injury, when we see a CT image of patients with osteophytes. Moreover, a traumatic examination, such as CT, should not be the first choice in the diagnosis of patients with TTAI in doubt.

## Patient consent

The legal guardians have provided informed consent for the publication of his case history.

## Data availability statement

All relevant data on this case is contained within the article.

## CRediT authorship contribution statement

**Kazuyuki Miyamoto:** Writing – original draft, Conceptualization. **Mako Sakakibara:** Conceptualization. **Hiroki Yamaga:** Data curation. **Atsuo Maeda:** Resources. **Masaharu Yagi:** Supervision. **Kenji Dohi:** Supervision.

## Declaration of competing interest

The authors declare that they have no known competing financial interests or personal relationships that could have appeared to influence the work reported in this paper.
